# Motivation moderates gender differences in navigation performance

**DOI:** 10.1038/s41598-023-43241-4

**Published:** 2023-09-25

**Authors:** Victor R. Schinazi, Dario Meloni, Jascha Grübel, Douglas J. Angus, Oliver Baumann, Raphael P. Weibel, Péter Jeszenszky, Christoph Hölscher, Tyler Thrash

**Affiliations:** 1 Chair of Cognitive Science, Department of Humanities, Social and Political Sciences, ETH Zurich, Zurich, Switzerland; 2 Department of Psychology, Bond University, Robina, Australia; 3 Future Health Technologies, Singapore-ETH Centre, Campus for Research Excellence and Technological Enterprise (CREATE), Singapore, Singapore; 4 Chair of Geoinformation Engineering, Department of Civil, Environmental and Geomatic Engineering, ETH Zurich, Zurich, Switzerland; 5 Chair of Technology Marketing, Department of Management, Technology and Economics, ETH Zurich, Zurich, Switzerland; 6 Centre for the Study of Language and Society, University of Bern, Bern, Switzerland; 7 Department of Biology, Saint Louis University, St. Louis, MO USA

**Keywords:** Human behaviour, Reward

## Abstract

Gender differences in navigation performance are a recurrent and controversial topic. Previous research suggests that men outperform women in navigation tasks and that men and women exhibit different navigation strategies. Here, we investigate whether motivation to complete the task moderates the relationship between navigation performance and gender. Participants learned the locations of landmarks in a novel virtual city. During learning, participants could trigger a top-down map that depicted their current position and the locations of the landmarks. During testing, participants were divided into control and treatment groups and were not allowed to consult the map. All participants were given 16 minutes to navigate to the landmarks, but those in the treatment group were monetarily penalized for every second they spent completing the task. Results revealed a negative relationship between physiological arousal and the time required to locate the landmarks. In addition, gender differences in strategy were found during learning, with women spending more time with the map and taking 40% longer than men to locate the landmarks. Interestingly, an interaction between gender and treatment group revealed that women in the control group required more time than men and women in the treatment group to retrieve the landmarks. During testing, women in the control group also took more circuitous routes compared to men in the control group and women in the treatment group. These results suggest that a concurrent and relevant stressor can motivate women to perform similarly to men, helping to diminish pervasive gender differences found in the navigation literature.

## Introduction

City dwellers often have to adapt to varying environmental and psychological situations during navigation, and the outcome is often uncertain. On the one hand, navigation may be interrupted by construction blocking a familiar route, which could cause one to miss an important appointment. On the other hand, one may be motivated to learn a novel and efficient route, which could lead that person to arrive at the appointment on time. In some situations, such as medical emergencies, the risk of navigating inefficiently may further reinforce the navigator's motivation to be punctual. Reactions to reinforcement may also differ depending on individual differences among navigators and their own expectations regarding their spatial abilities. In the present paper, we investigate the effects of motivation and gender on navigation efficiency in a virtual city.

Motivating efficient navigation with rewards may be regulated by simple arousal mechanisms. Indeed, the effects of prospective rewards on arousal have been consistently demonstrated in the reward processing literature^1,2^. Generally, the prospect of receiving a reward (or avoiding a loss or punishment) increases arousal, often enhancing cognitive task performance^3–5^. For example, response times in simple temporal judgment and target detection tasks are more accurate^6^ and faster^7,8^ when there is the prospect of a monetary reward. Similarly, self-reported and physiological measures of arousal are increased by cues that indicate a forthcoming reward relative to non-reward cues. In addition, reward cues typically enhance vigilance responses such as potentiated heart rate acceleration (initially) followed by sustained deceleration and increased skin conductance^2^. Notably, the prospect of a loss (and the possibility of avoiding that loss) can also lead to increased arousal to a greater extent than the prospect of a reward^9,10^. Previous research on the effects of a reward on navigation has generally focused on applying reinforcement learning principles to understanding navigation performance and strategies. Historically, researchers have considered the acquisition and use of spatial cues by rats during navigation as mere extensions of general reinforcement learning principles^11,12^. Similar to findings with rats, human navigators are capable of learning different navigation strategies simultaneously and can exhibit these strategies spontaneously^13^ or when reinforced^14^.

Research on human navigation distinguishes between place and response strategies. Place strategies rely on flexible spatial representations for finding one’s way towards a goal and are primarily associated with the hippocampus^15,16^. In contrast, response strategies are rigid associations between sequences of action and landmarks based in the dorsal striatum, specifically the caudate nucleus^17–19^. The selection of place or response strategies by human navigators is also affected by stress experienced before or during the retrieval of spatial information^20^. In these cases, stress can be understood as a specific type of motivation for the organism to return to psychological or physiological homeostasis. Strategy selection in response to acute stress during navigation is often adaptive in that it can prevent the stressor (e.g., social pressure, time pressure) from negatively affecting navigation efficiency^21,22^. While chronic stress may impair episodic memory in general by reducing the grey matter volume of the right hippocampus^23^, previous research has found that the dorsal striatum is less affected by over- or under-exposure to stress hormones^24^. Response strategies based on the dorsal striatum can allow the organism to avoid the negative effects of stress on retrieval during a short-term navigation task^24^. Consequently, human participants tend to rely more on familiar routes (an egocentric strategy) and less on shortcuts (an allocentric strategy) after exposure to acute stressors^21,25,26^.

Research on the effects of acute stress on navigation strategy and efficiency often focuses on stress experienced during retrieval in familiar environments^21,22,25^, but much of the stress typically associated with navigation occurs during encoding in somewhat novel environments^27^. According to the general memory literature, episodic memory encoding can be positively or negatively affected by acute stress^27^. The relationship between acute stress and encoding is most likely moderated by the delay between the stressor and the learning task and the relevance of the stressor for the learning task^27^. While stressors that occur exclusively before a learning task (e.g., Trier Social Stress Task, Cold Pressor Test) may positively^28^ or negatively^29^ affect encoding, less is known about stressors that are present during encoding (e.g., time pressure). At the same time, irrelevant information presented to an individual while performing a stressful task may not be learned as effectively. This effect is known in the general cognition literature as memory narrowing^30^. If the information being encoded is directly related to an emotionally engaging stressor, learning performance may be enhanced^31,32^.

Notably, the spontaneous selection of place and response strategies varies between men and women^13^, suggesting that gender may affect navigation differently depending on the specific type of stressor or task^33–35^. For example, Guenzel et al.^35^ asked male and female participants to complete two versions of a radial arm maze (spatial and non-spatial) and learn a route through an actual building. One week later, participants were asked to recall the route learned in each task. They found that a stressor presented before learning affected the performance of men on the non-spatial radial arm maze and the performance of women on the real-world navigation task. Similarly, Thomas and colleagues^34^ administered the Trier Social Stress test prior to asking participants to complete one of two versions of a virtual-navigation task. One version of the task had a visible search target and was considered landmark-guided navigation, while the other version of the task had an invisible search target and was considered cognitive-map-guided navigation. They found that, when the target was invisible, there was an interaction between gender and stress in which women under stress performed worse than all other groups of participants. Nonetheless, the interaction between gender and stress in the context of spatial navigation remains unclear. Indeed, Richardson and Vanderkaay Tomasulo^36^ found that men outperformed women on navigation-related pointing recall tasks and that stress negatively affected these tasks regardless of gender. However, these researchers did not find an interaction between gender and stress on navigation performance.

Spatial cognition research has a long history of investigating gender differences in navigation using both self-report and behavioral measures^37,38^. Men from various cultures (i.e., from the United States and Hungary) are more likely than women to report a strategy of orienting towards global reference points^39^. In contrast, women report attending to landmarks and route turns^40–42^. In addition, both genders report believing that men outperform women in navigation tasks^43^. These beliefs may partially underlie performance differences in navigation because spatial performance can generally be affected by self-perceived ability^44^.

Regarding behavioral measures, men have been found to navigate more efficiently and accurately than women in both real^45,46^ and virtual environments^47–52^. Specifically, men tend to perform better than women when the environment contains directional cues instead of positional cues^53,54^ and distal landmarks instead of proximal landmarks^52^. Similarly, men navigate more efficiently when given distance and direction information for a goal location, and women perform better when given information regarding landmarks and route turns^55^. Notably, men are as likely to rely on geometric cues compared to distal landmarks during navigation, but women rely more on distal landmarks than geometric cues^56^. Men are also better than women at recalling cardinal directions^57^, consistent with the observation that men tend to use cardinal directions more than women for orientation^38,41^. The predominant use of configurational and directional navigation strategies by men could also explain why they are more effective at using maps during navigation tasks^45,58,59^. These strategy differences may be related to idiosyncrasies in the ways each gender approaches spatial tasks. For example, women tend to spend more time wandering and pausing, while men tend to navigate faster and make riskier navigation decisions (e.g., taking shortcuts) even if not instructed to do so^33,47,60–63^. Women are also more sensitive to time pressure and show greater anxiety if speed is emphasized during a navigation task^64^. In addition, women demonstrate similar accuracy in spatial tasks compared to men but lower confidence levels if time pressure is removed^60^. Taken together, these findings emphasize the importance of considering differences in wayfinding behavior and emotional responses when assessing gender differences in spatial navigation.

For the present paper, we investigated the interaction between stress and gender and their effects on navigation performance. Specifically, participants searched for and learned six locations in a virtual environment and were then tested on their abilities to retrieve them in a particular order with or without time pressure. Time pressure was implemented by monetarily penalizing participants for every second spent during the task. We found that time pressure served as motivation to complete the navigation task more efficiently, especially for female participants. We interpret these results as one way to explain disparities in the literature on gender differences in navigation.

## Methods

### Participants

Sixty-nine participants were initially recruited via the University Registration Center for Participants (www.uast.uzh.ch). A power analysis indicated that this sample size was sufficient for detecting a medium to large effect size (*f* = 0.35, ɑ = 0.05, power = 0.81). The data from nine participants were excluded because of simulator sickness and technical issues with either the physiological equipment or the virtual reality environment that prevented them from completing the experiment. The data from sixty participants (29 female) were included in the final analyses. All of these participants were between 19 and 36 years of age (mean age = 23.33; SD = 3.12), psychologically healthy, and with normal or corrected-to-normal vision. Participants were randomly assigned to the control and treatment groups while attempting to keep the gender balanced between conditions. The two groups were not perfectly balanced for gender because of the participants that did not finish the experiment. There were 13 females in the control group (n_control_ = 31) and 16 females in the treatment group (n_treatment_ = 29). The experiment lasted approximately 60 minutes, and participants were compensated with CHF 40 regardless of their performance. Informed consent was obtained from all participants before the start of the experiment. All experimental protocols were approved by the ETH Zurich Ethics Commission (EK 2013-N-73) and the experiment was performed in accordance with the Declaration of Helsinki.

### Materials

#### Virtual environments

**Figure 1:**
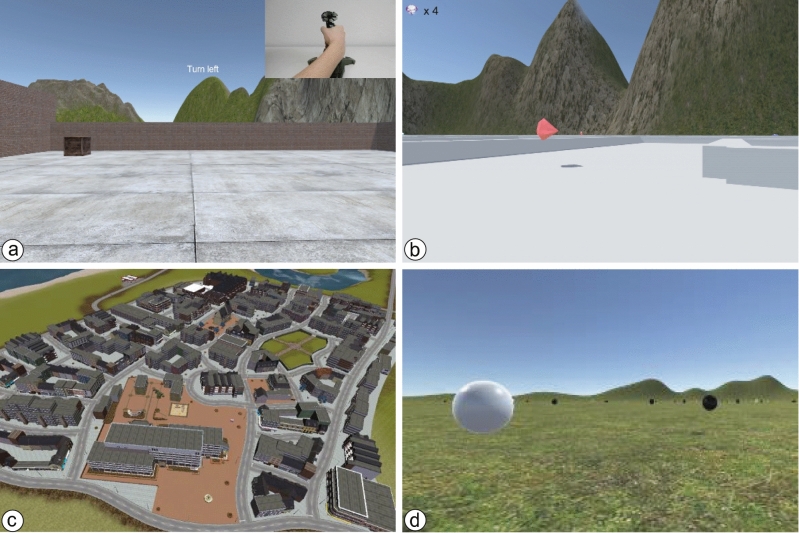
The four virtual environments used in the experiment: (**a**) Joystick training, (**b**) Maze, (**c**) City environment (from an oblique perspective), and (**d**) Sphere environment.

Four different virtual environments were created for the experiment (Fig. 1). The joystick training environment consisted of a small arena with two rooms and three scattered objects. The maze environment consisted of a circuitous path filled with scattered gems and separated by a series of walls. The sphere environment was a large open field surrounded by distant mountains and populated with white and black spheres. The city environment included 51 building blocks, three public squares, and a small park. Four target landmarks (i.e., museum, bar, city hall, and shop) were positioned in different areas of the city. The positions of the target landmarks were indicated with a white semicircle that floated above the ground, and the name of each landmark was written on a sign attached to the building’s front wall.

**Figure 2:**
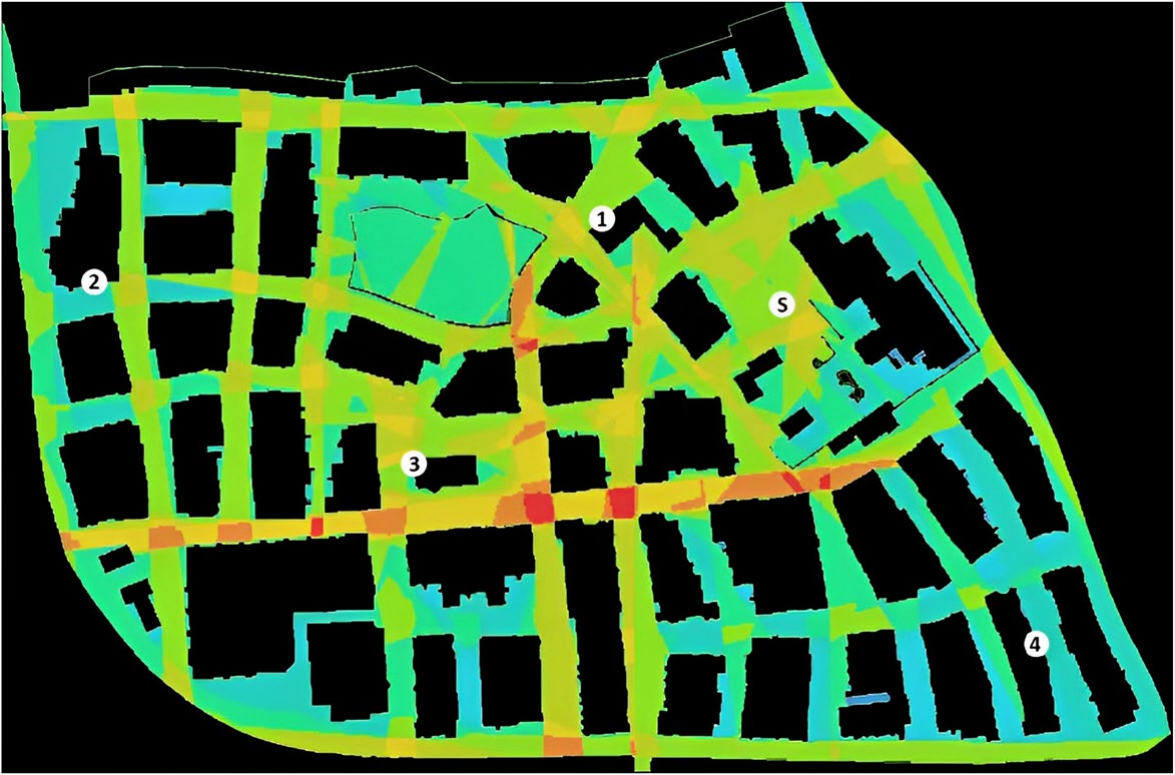
The visual integration map of the city that includes the positions of the four target landmarks and the starting location (S = Starting location; 1 = Museum; 2 = Bar; 3 = City hall; 4 = Shop). Warm colors reflect more visually integrated areas, while cool colors reflect less visually integrated areas.

To distribute the locations of the four target landmarks in the city, we conducted a space syntax analysis^65^. A Computer-Aided Design (CAD) map of the city was used to distinguish between the spaces that could be navigated (e.g., streets, alleys, and public squares) and the spaces occupied by buildings and barriers. Next, we performed a Visibility Graph Analysis (VGA)^66^ using Depthmap X^67^ to calculate the global visibility indices for different areas of the city (Fig. 2). We chose global visibility because participants were told to consider all parts of the city as potential locations for the target landmarks. As such, global visibility provided an index of the inter-visibility between the different spaces in the city. Here, higher scores (red regions) indicate areas that are more visibly accessible and potentially easier to find during a search task.

In contrast, lower scores (blue regions) indicate areas with lower visual access that may be more difficult to find. Given that participants were asked to search for locations in a specific order during the testing phase, we balanced the positions of the four target landmarks among areas in the city with high and low scores of visual integration. Two additional criteria guided the positioning of the four target landmarks. First, the four target landmarks needed to be distributed to cover as much of the city as possible. Second, we distributed the four target landmarks among locations with high and low visual integration such that the absolute difference in visual integration between two consecutive destinations was of a similar value along the route.

#### Hardware

The experiment was conducted using a high-performance computer (Core i7-3820 at 3.6 GHz; 12 GB of RAM; Nvidia Quadro K4000 with 3 GB RAM; Windows 10) with a 55″ ultra-high-definition television (Samsung Electronics UE55F6500). Participants used a joystick (Cyborg V.1 Flight Stick, Mad Catz) to navigate the virtual environments in the forward, backward, left, and right directions. Participants could also tilt their wrists to rotate left and right during navigation. The trigger on the front of the joystick was used to activate a map during the learning phase of the experiment.

Physiological data were collected based on the protocol described by Weibel and colleagues^68^. Specifically, electrodermal (EDA) and electrocardiogram activity (ECG) were collected using a PowerLab 8/35 recording device with FE116 GSR Amp and FE132 Bio Amp signal amplifiers (https://www.adinstruments.com). For electrodermal activity, electrodes were attached to the middle phalanges of the index and ring fingers of the non-dominant hand without pretreatment of the skin. For ECG, three electrodes were placed following the guidelines proposed by Stern and colleagues^69^. Specifically, two electrodes were placed on the second intercostal space below the middle of the clavicle for each side of the chest, and a third electrode was placed below the left rib cage. EDA and ECG were recorded at a rate of 1000 Hz.

#### Software

The experiment was deployed using a pre-release version of the Experiments in Virtual Environments (EVE) framework^70^. The EVE framework is based on the Unity game engine (https://unity.com) and allows researchers to setup, execute, and analyze data collected in desktop virtual reality experiments. We used LabChart 8.14 (https://www.adinstruments.com) to collect EDA and ECG data. Heart rate data were analyzed using LabChart's Heart Rate Variability (HRV) Module. EDA was analyzed using Matlab R2017a (https://www.mathworks.com) and Ledalab 3.4.9 (http://www.ledalab.com). All inferential statistics were conducted using RStudio 1.2.5033 (https://rstudio.com) and Jamovi (https://www.jamovi.org). Robust inferential statistics were based on the R package WRS2^71^, and spatial statistics were conducted using the R package evertools^72^. The evertools package is a companion software to EVE that facilitates processing of data collected in the framework. We used the ks package^73^ for two-sample comparisons of multivariate data,

#### Questionnaires

Participants were asked to complete a series of questionnaires before and after navigating the virtual environments. At the start of the experiment, participants completed demographic and gaming experience questionnaires followed by the Santa Barbara Sense of Direction Scale (SBSOD)^74^ and the first part of the Short Stress State Questionnaire (SSSQ)^75^. At the end of the experiment, they completed a Simulator Sickness Questionnaire (SSQ)^76^ and the second part of the SSSQ.

##### Demographics and gaming frequency

In the demographic questionnaire, participants reported their age, gender, level of education and employment status. The demographics questionnaire also included a series of questions to ensure that participants were not taking any psychoactive drugs and abstained from caffeine, tobacco, alcohol, and exercise for at least three hours before the experiment. The gaming questionnaire asked participants to report handedness, frequency of gaming, and type of control interface (i.e., joystick or mouse and keyboard) that they typically use when gaming.

##### Santa Barbara sense of direction scale

The SBSOD is a 15-item self-report scale of environmental spatial ability. For each item, participants are asked to report their level of agreement with a series of statements (e.g., "I am very good at judging distances") on a 7-point Likert Scale. A final score is then computed by calculating the average score such that higher scores indicate better perceived sense of direction.

##### Simulator sickness questionnaire

The SSQ consists of sixteen items that attempt to capture the various symptoms associated with simulator sickness. Participants were asked to report (0 = none; 1 = slight; 2 = moderate, and 3 = severe) the extent to which they experienced each symptom after navigating the three trials in the virtual environment. Data collected from this questionnaire is used to calculate three symptom cluster scores (i.e., disorientation, nausea, and oculomotor) and a total severity score.

##### Short stress state questionnaire

The SSSQ is a shorter version of the Dundee Stress State Questionnaire^77^ that distinguishes between distress, task engagement, and worry. The SSSQ is administered before and after participants engage in the experimental task. A difference score is calculated for each dimension by subtracting the pre-scores from the post-scores. The SSSQ was eventually excluded from the analysis because of an error in administering the survey.

### Procedure

Upon arriving at the laboratory, participants were briefed about the experiment and asked to read the information sheet that contained a short introduction to the experimental procedure and sign the consent form. Participants were asked to sit at a desk 1.6 m away from the monitor. The experimenter then attached and calibrated the EDA and ECG electrodes and dimmed the light in the room. The equipment used to collect physiological data was placed on a side table so that it could be adjusted to the left or right of the participants, depending on their dominant hand. Two movable walls were positioned to the sides of the desk to avoid any visual distractions. During the experiment, participants were asked to rest their non-dominant hand on the desk while manipulating the joystick. Participants were also instructed to remain still and avoid crossing their legs throughout the experiment.

After setting up the physiological recording equipment, participants completed the demographics questionnaire, the gaming questionnaire, the first part of the SSSQ, and the SBSOD. At this stage, participants were also told that they could abort the experiment without any consequences should they experience simulator sickness while navigating the virtual environments. Instructions for all phases of the experiment were presented via information screens before the start of each phase. Participants completed a joystick training phase followed by a physiological baseline phase. The main experiment consisted of learning and testing phases. Participants repeated the learning and testing phases over three trials.

During the joystick training phase, participants were shown a video depicting different joystick maneuvers (e.g., move forward, rotate right) and asked to reproduce them until they could successfully collect three objects and exit a virtual arena. In the physiological baseline phase, participants were placed inside a virtual maze and asked to follow a path indicated by arrows and collect a series of gems floating above the ground. The maze environment was deliberately designed so participants would relax and become familiar with the control interface. Physiological data collected during navigation in the maze environment was used to normalize data from the learning and testing phases of the experiment.

**Figure 3:**
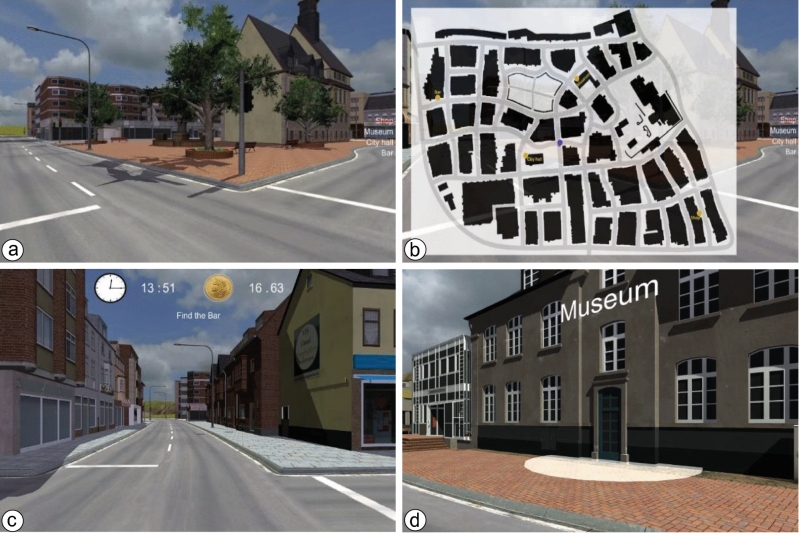
Screenshots of the learning and testing phases. (**a**) The city environment with the names of the four target locations. (**b**) The pop-up map with the current position of the participant (blue dot) and the positions of the four target locations (yellow circles) in the learning phase. (**c**) The testing phase showing the target destination, remaining time, and reward. (**d**) A close-up of a target destination.

Participants completed three navigation blocks, each consisting of a learning phase and a testing phase (see Fig. 3). Both learning and testing phases began at the same starting location. During the learning phase, participants were asked to freely explore the virtual city and learn the positions of four target locations. In order to facilitate learning, participants could press the trigger button on the joystick to activate a top-down map of the virtual city that showed their current location and the positions of the four target locations. This map was updated as participants moved through the environment such that it would always show their current location relative to the targets. A list with the names of the target locations was visible on the right side of the screen. Once participants arrived at a target location, a popup text acknowledged their arrival (e.g., "You have reached the City Hall"), and the name of the location was crossed out from the list (but remained visible on the map). Participants were not given a time limit to find the target locations but were told that they should take the time to learn their positions because they would be asked to navigate to them without the help of the map in the subsequent phase. During the learning phase, we recorded the time taken to find the four target locations and the total distance traveled. We also recorded the number of times the map was triggered and the amount of time that participants spent looking at the map.

During the testing phase, participants were randomly assigned to either the control or treatment group and asked to navigate to the four target locations in a specific order (i.e., Museum, Bar, City Hall, and Shop). Participants in both groups were also presented with icons of a clock and a coin at the top-center of the screen. The text next to the clock icon displayed the amount of time that remained for participants to complete the testing phase (in minutes and seconds). The countdown started at 16 min, and the task automatically ended when the clock reached zero. The text next to the coin icon displayed the amount of money participants would be rewarded at the end of the experiment. For participants in the control group, this value remained fixed at 10 CHF for each trial. For participants in the treatment group, the monetary reward started at 19.20 CHF and decreased at a pace of two centimes (i.e., two-hundredths of a Swiss Franc) per second. The parameters for the time and reward were established during pilot testing. The testing phase was completed once participants reached the four target locations in the correct order. Importantly, this stress manipulation relied on the difference in the monetary penalty distributed over time since both groups were presented with the time remaining to complete the task. Similar to the learning phase, we recorded the time taken to find the four target locations and the total distance traveled.

In order to ensure that participants in both groups were fairly compensated, they were asked to complete an additional task for compensation after the third block. Specifically, participants were immersed in the sphere environment and asked to collect only black spheres among several white spheres. Participants were compensated with 1 CHF for each collected sphere until they reached a total of 40 CHF. At the end of the experiment, participants were asked to complete the SSQ and the post version of the SSSQ.

### Design and analysis

We employed a 2 (motivation: control versus treatment) by 2 (gender) between-subjects design with eight dependent variables. Two physiological measures (i.e., heart rate and EDA) were used as manipulation checks. We also included three self-report measures: the SBSOD, SSQ, and a video game frequency questionnaire. Our four performance measures included the number of times the map was called during learning, time spent viewing the map, time to complete the learning phase, and time to complete the testing phase.

Heart rate was analyzed using the heart rate variability module within LabChart. The settings for human ECG were used for beat classification (i.e., RR interval of 400–1500 ms; complexity between 1 and 1.5). Extreme outliers were visually inspected and manually excluded if they were incorrectly classified as a beat. HRV analysis was not performed because there were large variations in task completion times (range 156–962 s) among participants. Indeed, 58% of participants required less than the 5 min recommended for HRV analyses to complete each trial of the testing phase^78,79^.

EDA was first exported from LabChart to LedaLab. In LedaLab, we downsampled the data from 1000 to 10 Hz and used Continuous Decomposition Analysis to extract the number of non-specific skin conductance responses (nSCR). For this analysis, we used a minimum amplitude threshold of 0.05 μs^80^. We disentangled the arousal associated with navigation decision-making from the arousal associated with steering through the maze environment with the control interface by subtracting nSCR and HR during the training phase from nSCR and HR during the learning and testing phases. We denote these differences in nSCR and HR as ΔnSCR and ΔHR, respectively.

Before conducting inferential statistics, we checked whether our data violated the normality and homogeneity of variance assumptions of ANOVA. Because normality was violated for each dependent measure except ΔHR, we elected to run two-way robust ANOVAs for the medians of each dependent measure rather than the standard two-way ANOVAs using means^71,81^. We also computed the Spearman's correlation between ΔHR and the time taken to complete the testing phase.

For the spatial analysis, we created four density distributions, each representing one condition of the experiment. First, participants' paths were aggregated over all three test trials and then combined over participants within the same group. We binned locations along these paths using a 90 × 90 grid and normalized to obtain comparable 3D distributions (i.e., in terms of x-coordinates, y-coordinates, and densities). The grid size was a function of environmental structure and needed to be determined such that features of interest could be detected without dominating the density. To assess differences among these four 3D distributions, we used a closed-form, nonparametric, asymptotically normal, density-based framework^73^. Following Duong and colleagues^82^, we compared these distributions using a KDE test^83^ with the R package ks^73^. Following Anderson and colleagues^84^, a discrepancy measure was used to compare intrasample differences to intersample differences^82^. To correct for multiple comparisons, we applied the Benjamini–Hochberg correction^85^. This type of correction reduces false discovery rates by applying an increasing penalty depending on the rank of the p-values from highest to lowest.

## Results

**Figure 4:**
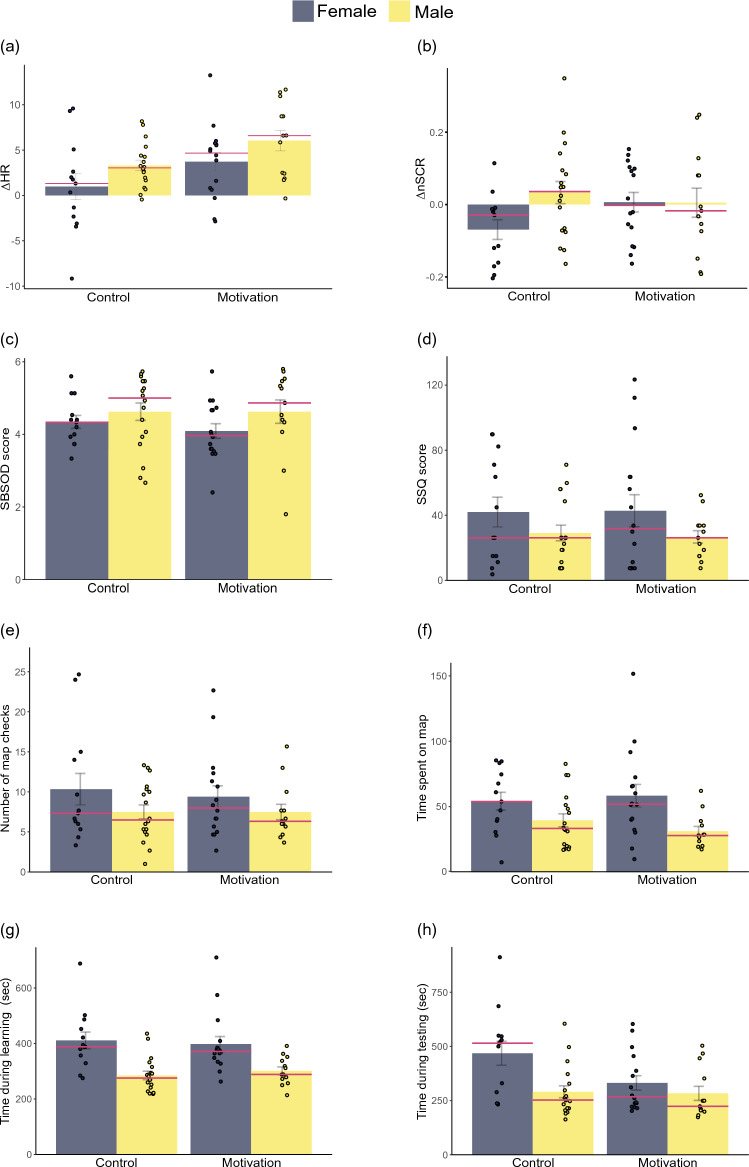
Graphs representing treatment and gender differences for (**a**) ΔHR, (**b**) ΔnSCR, (**c**) SBSOD scores, (**d**) SSQ scores, (**e**) number of map checks, (**f**) time spent on the map, (**g**) time during learning, and (**h**) time during testing. Dots represent individual data points, the bar height represents the mean data point, the red bars represent the median data points, and the error bars represent plus/minus one standard deviation.

Figure 4 presents the results of our analyses. The 2 (motivation) × 2 (gender) robust ANOVA for ΔHR revealed that participants' ΔHR was significantly higher (*Q* = 4.00, *p* = 0.045) in the treatment group (median = 5.11) than the control group (median = 2.57). There was no main effect of gender on ΔHR (*Q* = 1.12, *p* = 0.290) or interaction between gender and motivation on ΔHR (*Q* = 1.65, *p* = 0.199). The 2 × 2 robust ANOVA for ΔnSCR did not reveal a main effect of motivation (*Q* = 0.05, *p* = 0.817), a main effect of gender (*Q* = 0.19, *p* = 0.660), or an interaction between motivation and gender (*Q* = 0.56, *p* = 0.454).

The 2 × 2 robust ANOVA for SBSOD scores revealed that self-reported sense of direction was significantly higher (*Q* = 4.47, *p* = 0.034) for men (median = 4.93) than for women (median = 4.07). There was no main effect of motivation on SBSOD scores (*Q* = 0.45, *p* = 0.500) and no interaction between motivation and gender on SBSOD scores (*Q* = 0.30, *p* = 0.583). The 2 × 2 robust ANOVA for SSQ did not show a main effect of motivation (Q = 0.08, p = 0.774), a main effect of gender (*Q* = 0.08, *p* = 0.774), or an interaction between motivation and gender (*Q* = 0.08, *p* = 0.772). Similarly, the 2 × 2 robust ANOVA for the gaming frequency questionnaire did not show a main effect of motivation (Q = 0.00, p > 0.999), a main effect of gender (Q = 0.00, p > 0.999), or an interaction between motivation and gender (Q = 0.00, p > 0.999). Indeed, the medians of gaming frequency for all four groups were the same.

The 2 × 2 robust ANOVA for the number of times that the map was called during learning did not reveal a main effect of motivation (*Q* = 0.01, *p* = 0.914), a main effect of gender (*Q* = 0.29, *p* = 0.589), or an interaction between motivation and gender (*Q* = 0.19, *p* = 0.666). The 2 × 2 robust ANOVA for time spent viewing the map revealed that map time was significantly higher (*Q* = 5.62, *p* = 0.018) for women (median = 52.3 s) than for men (median = 31.1 s). For map time, there was no significant main effect of motivation (*Q* = 0.15, *p* = 0.696) or an interaction between motivation and gender (*Q* = 0.13, *p* = 0.718).

The 2 × 2 robust ANOVA for the time to complete the learning phase revealed that learning time was significantly higher (*Q* = 10.49, *p* = 0.001) for women (median = 380.0 s) than for men (median = 287.0 s). For learning time, there was no main effect of motivation (*Q* < 0.01, *p* = 0.960) or an interaction between motivation and gender (*Q* = 0.50, *p* = 0.479). The 2 × 2 robust ANOVA for time to complete the testing phase revealed an interaction between motivation and gender (*Q* = 4.30, *p* = 0.038). Specifically, women in the control group (median = 515 s) required more time to complete the testing phase than women in the treatment group (median = 267 s), men in the control group (median = 252 s), and men in the treatment group (median = 223 s). For testing time, there was also a significant main effect of motivation (*Q* = 5.23, *p* = 0.022) and a significant main effect of gender (*Q* = 6.43, *p* = 0.011). Specifically, participants in the control group (median = 289 s) required more time to complete the testing phase than participants in the treatment group (median = 249 s). Women (median = 393 s) also took longer than men (median = 288 s) to complete the testing phase. Finally, we found a significant Spearman correlation between ΔHR and time to complete the testing phase (*rho* = − 0.46, *p* < 0.001).

**Figure 5:**
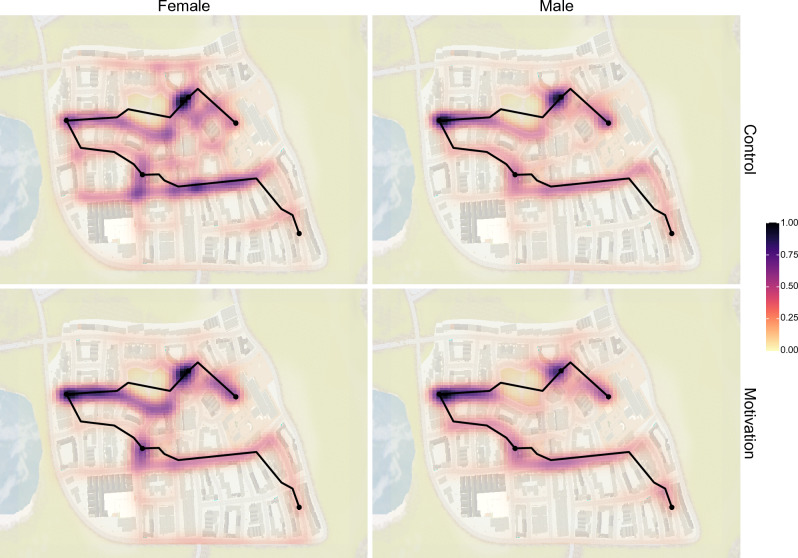
Normalized density of participants' paths using a 90 × 90 binning. The optimal shortest path is shown as a black line, and the destinations are shown as black points. The data are split by gender and treatment condition. A darker shade of red and higher opacity represent higher density. Because density is normalized, the black shade (1.00) corresponds to the location through which participants most often moved. Notably, female participants in the control condition produced very different density distributions compared to female participants in the motivation group and male participants in the control group.

A total of six KDE tests were used to compare density distributions between each pair of conditions from our 2 (gender) × 2 (treatment) design. For each of these comparisons, we report the corrected *a* and the p-value. The p-values that were lower than the corrected ɑ indicate that the two distributions were significantly different from each other. See Fig. 5 for a visualization of these density distributions. For women, control and motivation groups were significantly different (*a* = 0.0167, *p* = 0.0028). For men, control and motivation groups were not significantly different (*a* = 0.0417, *p* = 0.5584). For the control group, men and women were significantly different (*a* = 0.0083, *p* = 0.0008). For the motivation group, men and women were not significantly different (*a* = 0.0250, *p* = 0.0800). In addition, women in the motivation group were not significantly different from men in the control group (*a* = 0.0333, *p* = 0.0546). Despite our expectations, women in the control group were not significantly different from men in the motivation group (*a* = 0.0500, *p* = 0.9765).

## Discussion

To summarize, this study investigated the potential interaction between gender and a stress treatment that occurred during a navigation search task in VR. The key finding of this study is that women in the control group were slower to complete the testing phase than women in the treatment group or men in general. In addition, we found that women had lower self-reported sense of direction (SBSOD), spent more time viewing the map in the learning phase, and required more time to complete the learning phase compared to men. Supporting our manipulation of stress, we found a main effect of the treatment group on ΔHR and a negative correlation between ΔHR and time to complete the testing phase. Our spatial analyses of the routes taken during the testing phase revealed that women in the control group traveled along different routes than women in the treatment group or men in the control group. Taken together, these results suggest that, in a navigation context, differences between men and women may be partially explained by motivation to complete the task.

Consistent with previous research^37,38^, we found significant gender differences in navigation strategies. In general, women appear to be more cautious during the learning phase of the experiment, during which they spent more time consulting the map and learning the locations of the different landmarks. According to our spatial analyses, women in the control group also moved less directly towards the goals. This finding aligns with previous research that has shown that women tend to pause more often and navigate less directly while searching for a goal^33,47,60–63^. Gender differences in strategy selection may be connected to lower confidence in their ability to navigate, as evidenced by their lower SBSOD scores. Indeed, women have been found to rate their own sense of direction lower compared to men^86–88^. However, these main effects of gender may be superseded by other contextual variables, such as motivation to complete the navigation task.

In the present study, we found that motivation to complete the navigation task moderated the effect of gender on navigation performance, suggesting that common gender differences may be more nuanced than they appear. While some studies have assessed potential interactions between gender and stress treatments^34–36^, these studies found interactions in which stress was detrimental to the spatial performance of one or both genders. Specifically, Thomas et al.^34^ found that their stress treatment negatively affected the performance of women in a cognitive-map-guided navigation task (i.e., searching for an invisible target). Similarly, in Guenzel et al.^35^, stress negatively affected the accuracy of women in a real-world navigation task (i.e., recalling a route through a university building). Interestingly, these authors found that stress only negatively affected the accuracy of men in a non-spatial radial arm maze task. However, Richardson and VanderKaay Tomasulo^36^ did not find a stress by gender interaction for a spatial pointing recall task. Notably, all of these studies on gender and stress during navigation employed a stressor that occurred before learning and was unrelated to the spatial task.

The relationship between stress-induced cortisol and performance on spatial tasks can be affected by the amount of time between the stressor and the task^24^. According to Wiegert et al.^89^, cortisol can lead to the enhancement of Long-Term Potentiation (LTP) in the hippocampus at first, but over time (approximately 60 min), cortisol gradually begins to impair LTP. In humans, the threat of electric shock has been found to affect cortisol levels during goal-directed navigation without negatively affecting performance^25^. Brown et al. also found that cortisol was related to a decrease in shortcutting, suggesting that higher cortisol may have been related to less reliance on the hippocampus. One possible explanation for this discrepancy between navigation performance and apparent strategy selection may be the context of the stressor beyond the timing of the stressor per se. Critically, there is a strong tendency in spatial navigation research with humans to employ stressors that are unrelated to the navigation task, including electric shock^25^, the cold pressor test^28,35,90^, the Trier Social Stress Test^22,29,34^, the Paced Auditory Serial Addition Task^26^, and the Mirror Tracing Task^36^.

One notable exception to this pattern are the results from Brunye et al.^21^. In their experiment, participants first performed a search task for 20 landmark buildings in a particular order in a large virtual environment and were then asked to search for these same buildings in order under low, medium, or high time pressure. Similar to Brown et al.^25^, Brunye et al.^21^ found that more stress led to a shift from map-based to route-based strategies. They also found that time pressure negatively affected search accuracy. Interestingly, their individual differences analyses indicated that the tendency to adopt a map-based strategy was best explained by video game experience. In contrast, our results indicate that time-related monetary penalties positively affected navigation performance, especially for women. Future work should consider the potential interaction between the timing and relevance of a stressor for navigation to further disentangle these results.

An alternative explanation for our results is that there was a ceiling effect on testing performance in which either gender could have performed better than the other on a more difficult task. Here, participants only searched for six landmarks which is considerably less than some other studies^21^. However, this alternative does not explain the difference we observed in the routes taken by women in the control and motivation groups. Another possible limitation is our use of virtual reality for inducing and measuring stress during navigation. While we acknowledge that that are clear differences between virtual and real-world navigation^91^, there is a growing body of evidence that navigation through virtual environments represents at least a subset of real-world navigation behavior^92–95^. Despite these possible limitations, the present findings point towards an explanation for gender differences in navigation behavior that has so far been largely overlooked.

## Conclusion

The present paper provides evidence for an alternative interpretation of the pervasive gender differences found in the spatial cognition and navigation literature. While previous research has emphasized the detrimental effects of stressors experienced during encoding rather than testing, we found that women performed similarly to men when motivated to complete the navigation task with a concurrent and relevant stressor. These findings are important because, rather than providing evidence for or against gender differences, they elucidate some conditions in which gender differences occur. We also characterized the physiological correlates and spatial behaviors of stress and motivation during navigation. Towards this end, we demonstrated a correlation between change in heart rate and time required to complete the task, as well as differences between women in the motivated and control groups in terms of map use and path choice. These findings may be especially pertinent for understanding everyday navigation for which gender differences may have been exaggerated.

## Data Availability

Data and additional online materials are openly available on the project’s Open Science Framework page (https://osf.io/d9csb/). We have no conflicts of interest to disclose. This project was funded by the Chair of Cognitive Science startup grant. We thank Tarn Duong for pointing us to the Benjamini–Hochberg correction used to compare our multivariate data and providing a reference for the implementation. We thank VIS Games for providing the model of the city used in this study.
